# Therapeutic Application of Drug-Conjugated HER2 Oligobody (HER2-DOligobody)

**DOI:** 10.3390/ijms21093286

**Published:** 2020-05-06

**Authors:** Hyun Jung Kim, Ho Jin Sung, Yul Min Lee, Sun Il Choi, Yun-Hee Kim, Kyun Heo, In-Hoo Kim

**Affiliations:** 1 Research Institute, National Cancer Center, 323 Ilsan-ro, Ilsandong-gu, Goyang 10408, Korea; hjungkim@ncc.re.kr (H.J.K.); shjbleu11@gmail.com (H.J.S.); 74911@ncc.re.kr (Y.M.L.); sunil@ncc.re.kr (S.I.C.); sensia37@ncc.re.kr (Y.-H.K.); 2 Department of Bioinspired Science, Ewha Womans University, 52 Ewhayeodae-gil, Seodaemun-gu, Seoul 03760, Korea; 3 Research Institute, JP Bio A Co., 302 Galmachi-ro, Jungwon-gu, Seongnam 13201, Korea; 4 Department of Cancer Biomedical Science, Graduate School of Cancer Science and Policy, 323 Ilsan-ro, Ilsandong-gu, Goyang 10408, Korea; 5 Biopharmaceutical Chemistry Major, School of Applied Chemistry, Kookmin University, 77 Jeongneung-ro, Seongbuk-gu, Seoul 02707, Korea

**Keywords:** aptamer, antibody-drug conjugate (ADC), oligobody, drug-conjugated oligobody (DOligobody), HER2, cancer therapeutics

## Abstract

Antibody drug conjugates (ADCs), consisting of a cancer-specific antibody and cytotoxic payload, are shown to be a potent class of anticancer therapeutics, with enhanced therapeutic efficacy and reduced “off-target” side effects. However, the therapeutic window of ADCs is narrowed by problems such as difficulty in site-specific conjugation of payload, changes in antibody stability due to payload conjugation, and difficulty in tissue penetration. In this respect, aptamers have advantages in drug-delivery, as they can be easily and stably conjugated with cytotoxic drugs. We previously reported that oligobody, an aptamer-antibody complex, is a novel delivery method for aptamer-based therapeutics. In the current study, we describe DOligobody, a drug-conjugated oligobody comprising an aptamer-drug conjugate and an antibody. A cotinine-conjugated anti-HER2 aptamer (cot-HER2apt) was specifically bound to HER2-positive NCI-N87 cells, and underwent receptor-mediated endocytosis. Further, HER2-DOligobody, a cot-HER2apt-conjugated monomethyl auristatin E (cot-HER2apt-MMAE) oligobody, inhibited the growth of HER2-positive NCI-N87 cells. Finally, systemic administration of HER2-DOligobody significantly reduced tumor growth in a xenograft mouse model. Taken together, these results suggest that our DOligobody strategy may be a powerful platform for rapid, low-cost and effective cancer therapy.

## 1. Introduction

Cytotoxic chemotherapies inhibit cell division and are being widely used for various types of cancer [1]. However, in addition to cancer cells, cytotoxic drugs also reach essentially all other cells throughout the body and cause toxicity. This consequently causes adverse side effects, such as hair loss, fatigue, diarrhea, nausea and vomiting, skin rashes, and oral ulcerations [2]. Since the mid-1990s, targeted therapies, that may minimize side effects and effectively inhibit cancer by specifically targeting only cancer cells, have emerged as important means of disease management for patients with cancer. Among the targeted therapies developed over the last 25 years, monoclonal antibodies (mAbs) and antibody-based therapeutics have provided a promising strategy for cancer therapeutics [3]. To date, there have been more than 30 Food and Drug Administration (FDA)-approved mAbs, with more than 600 mAbs currently being tested in clinical trials of cancer therapeutics [4,5].

Antibody-drug conjugates (ADCs) are one type of antibody-based therapeutics, and are composed of target-antigen specific mAbs conjugated with cytotoxic drugs (payload) through chemical linkers. The antibody portion of ADCs binds to specific cell-surface antigens and the complex is then internalized through receptor-mediated endocytosis. The payload is consequently released from the complex in lysosomes and its function exerted in the cancer cells, such as inhibition of DNA replication or microtubule polymerization [6,7]. Due to their specific targeting of cancer cells, ADCs have lesser side effects than other cytotoxic agents and provide a wider therapeutic application. Currently, seven ADCs, gemtuzumab ozogamicin (Mylotarg^®^), brentuximab vedotin (Adcetris^®^), ado-trastuzumab emtansine (Kadcyla^®^), inotuzumab ozogamicin (Besponsa^®^), polatuzumab vedotin-piiq (Polivy^®^), Enfortumab vedotin (Padcev^®^), and Trastuzumab deruxtecan (Enhertu^®^), have received market approval as cancer therapies [8,9,10,11,12,13,14]. Nevertheless, due to the nature of antibodies, conjugation of the mAbs and payloads typically results in a mixture ADC with varied drug-to-antibody ratios (DARs), and increased ADC aggregation due to antibody surface changes. This results in decreased efficacy and lower overall stability of the ADCs [15,16,17]. Moreover, due to their relatively large size, it is difficult for ADCs to penetrate tumor vessels and permeate tumor tissue, reducing the overall amount of antibody molecules delivered internally to solid tumors. This may lead to acquired resistance by the cancer and subsequent treatment failure [18,19]. Therefore, new platform technologies are needed to overcome these challenges in the design and therapeutic use of ADCs.

Aptamers are single-stranded RNA or DNA oligonucleotides that bind a variety of targets, ranging from small molecules, to proteins, to whole cells [20,21,22]. Due to several significant advantages, such as greater stability, easier synthesis and lower production cost, aptamers have become attractive molecules for diagnostic and therapeutic applications [23,24]. However, a major disadvantage of aptamers is that they have low stability in vivo, and low pharmacokinetics when systemically injected [25]. For that reason, only one aptamer is currently administered by intravitreal injection for the treatment of age-related macular degeneration (AMD), that being pegaptanib sodium (Macugen^®^) [26]. In a previous study, we described the use of a monoclonal antibody as a universal aptamer-carrying vehicle, which we termed an “oligobody” (oligomer + antibody). The oligobody was developed as a reaction between an anti-VEGF aptamer, which is linked to a cotinine as a hapten, and an anti-cotinine antibody. We found that since an aptamer is a small molecule, it would easily penetrate tumor tissue after oligobody binding to the target receptor. In addition, the result of xenograft modelling showed that the pharmacokinetics of the aptamer were improved by the oligobody complex, and administration of the oligobody reduced tumor growth in vivo. Overall, the oligobody appeared to overcome the therapeutic limitations of antibodies with regards to tumor-penetrating ability, and the amount of circulating aptamer was significantly enhanced in vivo by complexation. These findings also support that the oligobody overcomes the disadvantages of the aptamer, and possibly facilitates the clinical application of the aptamer [27]. Therefore, the oligobody strategy may be a powerful delivery method for use in anti-cancer therapeutics.

In the current study, we developed a novel platform referred to as a “Drug-conjugate Oligobody” (DOligobody), which had monomethyl auristatin E (MMAE) conjugated at the 3′-end of the aptamer to enhance the potency of the oligobody. MMAE is an antimitotic agent that inhibits cell division by blocking tubulin polymerization [28,29]. For this proof-of-principle study, we used cotinine-conjugated anti-HER2 DNA aptamer sequences previously developed by Mahlknecht G. et al. [30]. The 14-mer aptamer and dimeric 28-mer aptamer, conjugated with cotinine, were designated as cot-HER2apt14 and cot-HER2apt28, respectively. We found that the cot-HER2apts showed specific binding and receptor-mediated endocytosis in HER2-positive cancer cells, and cot-HER2apt-MMAEs effectively inhibited cell viability. Moreover, a cot-HER2apt-MMAE oligobody (HER2-DOligobody) significantly reduced tumor growth in a xenograft mouse model, without causing severe toxicity. These results suggest that our DOligobody strategy may be applicable as an attractive anti-cancer therapeutic platform.

## 2. Results

### 2.1. Characterization of HER2 Aptamers and Cotinine- and MMAE-Conjugated HER2-Aptamers

**Figure 1:**
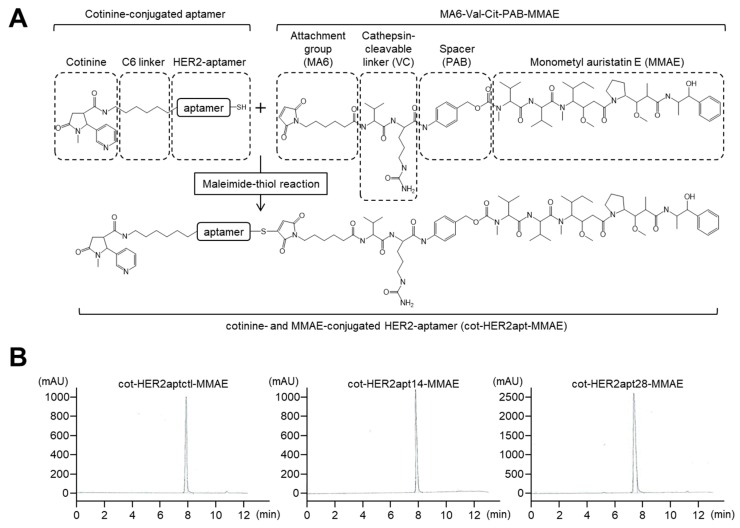
Synthesis of cot-HER2apt-MMAEs and their purification. (**A**) The cot-HER2apt-MMAEs consist of three main components: the cotinine-linker, HER2 aptamer, and MMAE-linker. The cotinine-conjugated HER2 aptamers (cot-HER2apts) were conjugated at the 3′-end with maleimidocaproyl-valine-citrulline-p-aminobenzoyloxycarbonyl-monomethyl auristatin E (MA6-VC-PAB-MMAE) through maleimide-thiol reactions. (**B**) Cot-HER2aptctl-MMAE, cot-HER2apt14-MMAE, and cot-HER2apt28-MMAE were purified to >98% purity by reverse-phase high-pressure liquid chromatography (C18 column).

To evaluate proof-of-principle of our DOligobody strategy, we used two DNA aptamers (HER2apt14 and HER2apt28), which were previously identified as specific aptamers against HER2 [30]. HER2apt14, HER2apt28 and HER2aptctl were linked to cotinine at the 5′-end with C6 linker, and linked to the microtubule inhibitor MMAE at the 3′-end with the cathepsin B cleavable linker MA6-Val-Cit-PAB. The HER2aptctl was used as a control of HER2-aptamers. Finally, cot-HER2apt-MMAEs were synthesized via a maleimide-thiol reaction (Figure 1A). HPLC analysis showed that each cot-HER2apt-MMAE was appropriately synthesized and purified to high purity (Figure 1B).

**Figure 2:**
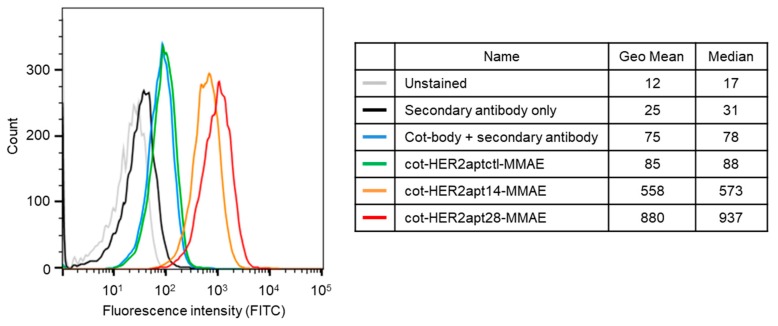
Flow cytometric analysis of cot-HER2apt-MMAEs. NCI-N87 cells (3 × 10^5^) were incubated with 100 pmoL of cot-HER2aptctl-MMAE, cot-HER2apt14-MMAE, or cot-HER2apt28-MMAE at 4 °C. The cells incubated with 100 pmoL of fluorescein isothiocyanate (FITC)-labeled secondary antibody (with or without primary antibody) served as negative controls. Fluorescence was measured by using a FACSVerse flow cytometer.

To evaluate whether the cot-HER2apt-MMAEs could bind to HER2-positive cancer cells, we performed FACS analysis using NCI-N87 human gastric cancer cells, which are known to be HER2-positive. After incubation with cot-HER2apt-MMAEs, the cells were stained with FITC-labeled anti-human secondary antibody, and their binding specificity was analyzed by FACS. Our results revealed that cot-HER2apt14-MMAE and cot-HER2apt28-MMAE interacted with HER2 expressed on the surface of NCI-N87 cells, whereas the cot-HER2aptctl failed to bind (Figure 2). The fold change in the mean fluorescence intensity (MFI) for cot-HER2apt14-MMAE and cot-HER2apt28-MMAE, compared to that for cot-HER2aptctl-MMAE, was determined as 6.7 and 10.4, respectively. The result demonstrated that the cot-HER2apt28-MMAE has more binding affinity for HER2 than cot-HER2apt14-MMAE. 

**Figure 3:**
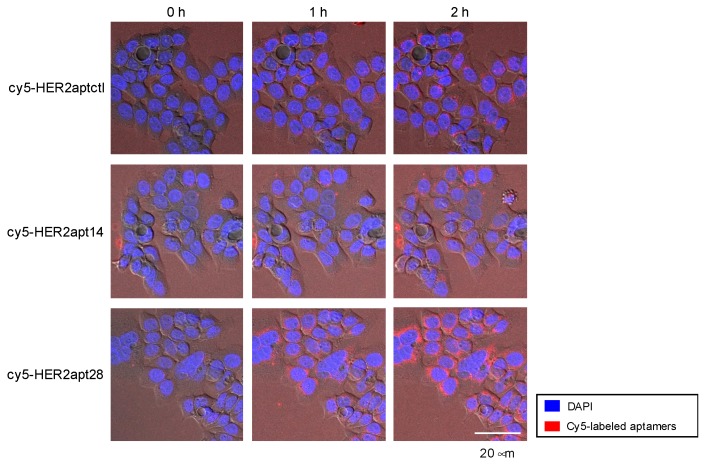
Confocal microscopic imaging of cy5-HER2apts internalization. NCI-N87 cells (3 × 10^5^) were treated with Cy5-labeled aptamers (cy5-HER2aptctl, cy5-HER2apt14, and cy5-HER2apt28) and DAPI, and incubated at 37 °C for 2 h. The representative are merged images of DIC, DAPI, and Cy5. Internalization of aptamers was visualized using an LSM 780 confocal microscope. Representative fluorescent images were obtained at 0, 1, and 2 h post treatment with cy5-HER2apts. Original magnification, 40×; Scale bar, 20 μm.

Receptor-mediated endocytosis is required for cytotoxic drugs, such as MMAE conjugated to aptamers, to be biologically active inside cancer cells, which is achieved via usage of the protease-labile dipeptide linker (valine-citrulline). To measure internalization of HER2apt14 and HER2apt28 into NCI-N87 cells, Cy5 fluorescent dye was conjugated to the 5′-end of the aptamers (cy5-HER2apt14 and cy5-HER2apt28), and endocytosis was monitored by confocal microscopy. Following exposure of cells to the cy5-HER2apt14 and cy5-HER2apt28, the Cy5 signal was gradually increased inside the cell. The representative images indicated time-dependent internalization of cy5-HER2apt14 and cy5-HER2apt28 into the cells (Figure 3). These results suggest that the HER2 aptamers specifically bound to surface HER2, and were internalized into the tumor cells via receptor-mediated endocytosis.

### 2.2. Antitumor Activity of cot-HER2apt-MMAEs In Vitro

**Figure 4:**
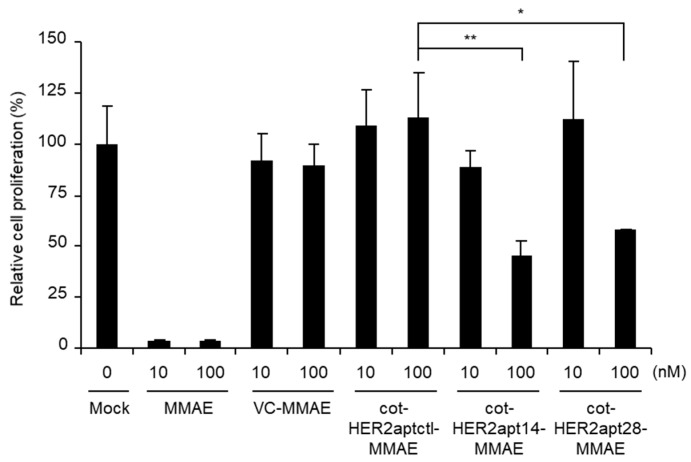
Cytotoxic effect of cot-HER2apt-MMAEs on NCI-N87 cells. The cells (1 × 10^4^) were treated for 6 d with 10 or 100 nM free monomethyl auristatin E (MMAE), valine-citrulline (VC)-MMAE, cot-HER2aptctl-MMAE, cot-HER2apt14-MMAE, or cot-HER2apt28-MMAE. Cell viability was assessed using WST-1 proliferation assays. Data are shown as the mean ± standard deviation (SD). The *p*-value was calculated using Student *t*-test (* *p* < 0.05 and ** *p* < 0.01).

The effect of cot-HER2apt-MMAEs on cancer cell viability was verified. NCI-N87 cells were treated with cot-HER2apt14-MMAE and cot-HER2apt28-MMAE at concentrations of 10 nM and 100 nM, and cell proliferation was evaluated by WST-1 analysis (Figure 4). Due to its strong cytotoxicity, MMAE cannot be used as a drug by itself, but has been applied with ADCs through conjugation with cleavable linkers and antibodies. In the current study, we found that cell proliferation was significantly inhibited by treatment with free MMAE, but proliferation was not reduced by treatment with VC-MMAE. We also found that cot-HER2apt14-MMAE and cot-HER2apt28-MMAE effectively decreased cell proliferation. Cell viability results demonstrated that the 50% inhibition concentration (IC_50_) of cot-HER2apt14-MMAE and cot-HER2apt28-MMAE were 83.68 nM and 168.1 nM, respectively. These results showed that cot-HER2apt-MMAEs effectively inhibited cell growth by targeting HER2-positive cancer cells and being internalized. Previously, Mahlknecht et al. [30]. reported that both monomeric aptamers (14 nucleotides) and trimeric HER2 aptamers (42 nucleotides) bind HER2, but only the trimeric aptamers inhibit growth of gastric cancer cells in vitro and in vivo. Here, we found that although the trimeric form of the aptamer (cot-HER2apt42-MMAE) bound more potently to HER2-positive cells than the monomeric form did (data not shown), cot-HER2apt14-MMAE was sufficient to inhibit tumor growth.

### 2.3. Antitumor Activity of HER2-DOligobodies In Vivo

**Figure 5:**
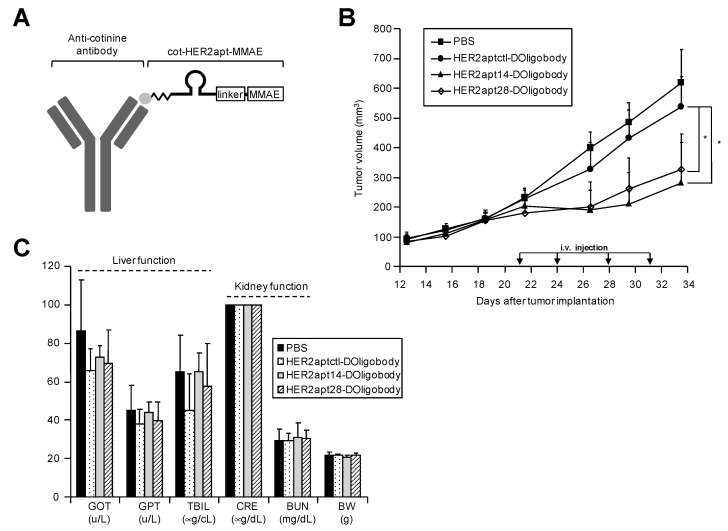
Anti-tumor activity of systemically administered HER2 DOligobodies in a mouse xenograft model. (**A**) HER2 DOligobody schematic representation. The DOligobody consists of the four elements: the cotinine (cot)-body, cot-linker, aptamer and monomethyl auristatin E (MMAE). (**B**) NCI-N87 cells (1 × 10^7^) were subcutaneously injected into the flank region of BALB/c nude mice. When the tumors reached 200 mm^3^, the mice (*n* = 10 per group) were intravenously injected with PBS (■), control HER2-DOligobody (●), HER2apt14-DOligobody (▲), or HER2apt28-DOligobody (◇) (1.27 mg/kg cot-HER2apt-MMAEs pre-incubated with 10 mg/kg cot-body). Tumor volumes were monitored for 34 d. Data are shown as the mean ± standard error of the mean (SEM); * *p* < 0.05 compared with the control HER2-DOligobody group, Student’s *t*-test. (**C**) In vivo toxicity reflects changes in body weight of the mice and serum concentrations of GOT, GPT, BUN, CRE and TBIL measured 36 d after tumor implantation. All the data represent the means ± SEM from three independent experiments. GOT, glutamic oxaloacetic transaminase; GPT, glutamic pyruvic transaminase; TBIL, total bilirubin; CRE, creatinine; BUN, blood urea nitrogen; BW, body weight.

We also examined the in vivo potential of HER2 DOligobodies on tumor growth, using a xenograft mouse model of human gastric cancer. Previously, we found that aptamers, which have low molecular weights, are cleared rapidly from the bloodstream when injected into blood vessels [27]. Therefore, we used HER2-DOligobody, which consists of cot-HER2apt complexed with anti-cotinine antibody, for in vivo experiments (Figure 5A). Gastric tumors were established in nude mice using NCI-N87 cells. We injected the cells (1 × 10^7^ cells) subcutaneously into the flank regions of BALB/c-nude mice, and monitored tumor growth. At 12 d post cell injection, tumor volumes had reached 200 mm^3^ and the animals were divided into four groups (*n* = 10 each experimental point). The animals were administered PBS as control, control HER2-DOligobody, HER2apt14-DOligobody or HER2apt28-DOligobody, by intravenous injection. Injection of HER2apt14-DOligobody or HER2apt28-DOligobody significantly reduced tumor growth (*p* < 0.05), whereas injection of control HER2-DOligobody had no such effect (Figure 5B). On the other hand, there was no noticeable difference in tumor growth between the groups treated with HER2apt14-DOligobody and HER2apt28-DOligobody. These findings indicate that systemic injection of DOligobody effectively inhibited tumor growth, and that the monomeric aptamer (cot-HER2apt14-MMAE) and multimeric aptamer (cot-HER2apt28-MMAE) had similar anti-cancer efficacies.

We also assessed the toxicity of the HER2-DOligobodies in the mice by monitoring liver and kidney function and changes in body weight. No significant changes were observed between the groups treated with the HER2-DOligobodies and the control (Figure 5C). These results suggest that HER2-DOligobodies did not induce severe toxicity in vivo.

## 3. Discussion

As candidates of targeted therapy for anti-cancer agents, mAbs and other antibody-based therapeutics are used as powerful anticancer agents, as they show high efficacy by specifically recognizing cancer [31]. Seven ADCs have received market approval so far and over 100 are being investigated in various stages of clinical trials. ADCs offer many advantages over traditional small molecule drugs and monoclonal antibodies themselves. Although ADCs are recognized as one of the most promising tools for the selective ablation of cancer cells, several critical issues must be addressed and investigated regarding the development of ADCs, including optimization of the linker, conjugation site, payload and drug loading [32].

Many macromolecules, such as antibodies, tend to be prone to conformational changes that may lead to the loss of their unique tertiary structures. This may result in misfolding aggregates and the loss of a large portion of the molecules during the manufacturing process, such as functional-group activation, conjugation, buffer exchange, purification, storage, or other steps. During the conjugation process of ADCs, side chains of cysteine, lysine, or keto groups of the carbohydrate of the monoclonal antibody, may be activated and also gain functionality from the linking of the payload [33]. Unfortunately, during the conjugation reaction, the activated intermediates may mediate inter-molecular crosslinking, resulting in aggregation. In addition, payload conjugation can also impact conformational changes of mAbs and alter their aggregation potentials. In contrast, short sequences of DNA/RNA aptamers exhibit good solubility (>150 mg/mL) in both aqueous buffers and solutions containing high concentrations of organic solvent [34]. Therefore, in the field of payload conjugation, aptamers are attractive molecules due to their attributes of high solubility, thermal and chemical stability, conformational reconfiguration, and low batch-to-batch variation.

Furthermore, the stoichiometry between mAbs and payloads cannot be entirely controlled. The resulting product may be heterogeneous mixtures of ADCs, containing 1–8 drug molecules per antibody (DAR1 to DAR8), and even containing some non-conjugated antibodies. Each ADC in the mixture may exhibit individual characteristics concerning pharmacokinetics, toxicology and efficacy. Too few of the drug molecules being attached to the antibody results in low efficacy, whereas too many of the payload molecules causes the ADC to have unstable properties, increased plasma clearance, reduced half-life, and increased toxicity [17]. Accordingly, another advantage of aptamers is that they are easy to chemically synthesize, and therefore the number of payloads conjugated at any desired site of aptamer can be precisely controlled [35]. This may reduce the work required during the chemical process to obtain homogenous payloads and increase the yield of the conjugates.

The relatively large size (~150 kDa) and long half-life characteristics of mAbs and antibody-based therapeutics are advantageous for therapeutic application, as they allow for longer time intervals for the drugs to act on tumor cells. However, the large size of mAbs makes it difficult for them to penetrate deeper inside tumors, and consequently their effect on solid tumors is often not as good as expected [18]. The smaller size of small-molecule drugs or aptamers should allow for more efficient extravasation and tumor penetration, and consequently show higher cancer therapeutic efficacy [36,37]. However, a drawback of the low molecular weights of aptamers is that they disappear rapidly from the bloodstream when injected into blood vessels, displaying short half-lives (<20 min) [20,38]. To take advantage of the characteristics of both aptamers and antibodies, we previously proposed the concept of oligobodies, which maintain the tissue-penetrating abilities and increased in vivo pharmacokinetics of aptamers. In that study, we showed that the complexation of oligobodies extends the in vivo half-life of aptamers. In our DOligobody system, the payload-conjugated aptamer, after separation from the antibody, is expected to penetrate deeply into tumor tissue, and thereby effectively increase the inhibition of tumor growth. Further studies would be needed to optimize the dose of DOligobody in the xenograft model. It might be possible to administer higher doses of DOligobody into mice, because we already validated that the DOligobody was non-toxic in vivo. For this reason, we expect that the administration of higher doses of DOligobody will further reduce tumor growth in vivo.

## 4. Materials and Methods

### 4.1. Cell Lines

Cell culture media was purchased from Thermo Scientific Hyclone (Waltham, MA, USA). Human breast cancer (SKBR3) and human gastric carcinoma (NCI-N87) cells were obtained from American Type Culture Collection (ATCC; Manassas, VA, USA). The cells were maintained in Roswell Park Memorial Institute (RPMI) medium supplemented with 10% heat-inactivated fetal bovine serum (FBS; Thermo Scientific Hyclone).

### 4.2. Synthesis of cot-HER2apt-MMAEs and HER2-DOligobodies

The cot-HER2apts were synthesized as described previously [27,39]. Briefly, HER2 aptamers were synthesized with an amino C6 linker at the 5′-terminus, and conjugated to cotinine using the active ester method. Next, the cot-HER2apts were purified via reverse-phase high-pressure liquid chromatography (HPLC) using an XBridge Prep C18 column (Waters, Milford, MA, USA). The purified cot-HER2apts were analyzed using ion-trap mass spectrometry with electrospray ionization (ESI-IT/MS; ST Pham Co., Seoul, Korea).

To conjugate cot-HER2apts with MMAE, cot-HER2apts bearing a 3′ thiol C3 S-S linker were reduced to generate a free thiol by incubating in 0.1 M triethylammonium acetate (TEAA) with 1 mM tris(2-carboxyethyl)phosphine (TCEP) at 70 °C for 5 min, followed by incubation at room temperature for 2 h. Excess TCEP was removed by exchanging into phosphate-buffered saline (PBS) with 2 mM EDTA, using an Amicon Ultra 3kD spin column (Millipore, Billerica, MA, USA). Maleimidocaproyl-valine-citrulline-p-aminobenzoyloxycarbonyl-monomethyl auristatin E (MC-Val-Cit-PAB-MMAE; Levena Biopharma, San Diego, CA, USA) was dissolved in dimethyl sulfoxide (DMSO) at a concentration of 20 mM, and added to the reduced cot-HER2apts in PBS with 2 mM EDTA at a 5-fold molar excess of drug. Excess MC-Val-Cit-PAB-MMAE was removed using Amicon Ultra 3kD spin columns. The cot-HER2apt-MMAEs were analyzed by reversed-phase analytical HPLC on a 4.6 × 50 mm XBridge C18 column, heated to 65 °C with eluents of 0.1 M TEAA at pH 7.0 (eluent A) and acetonitrile (eluent B). The following elution profile was utilized for analysis: 0–5 min, 90% eluent A and 10% eluent B, with eluent B increasing from 10–20% at a flow rate of 1 mL/min; then 5–15 min, eluent B increasing from 20–70% at a flow rate of 1 mL/min. Elution of the cot-HER2apt-MMAEs was monitored by UV absorbance at 260 nm.

The HER2-DOligobodies were prepared by mixing cot-HER2apt-MMAEs and the anti-cotinine antibody (cot-body) at a 2:1 molar ratio. The mixture was then incubated for 30 min at room temperature with gentle shaking.

### 4.3. Flow Cytometry (FACS)

To evaluate the binding capacity of the cot-HER2apt-MMAEs, FACS analysis was performed. Briefly, 100 pmol of each cot-HER2apt-MMAEs was incubated with NCI-N87 cells (3 × 10^5^) in binding buffer (5 mM MgCl_2_ in Dulbecco’s PBS supplemented with 0.1 mg/mL tRNA and 1 mg/mL bovine serum albumin) at 4 °C for 30 min. The cells were then washed three times with binding buffer containing 0.1 % NaN_3_, and incubated with 50 pmol of cot-body at 4 °C for 30 min. The cells were then washed three times, incubated with 100 pmol of fluorescein isothiocyanate (FITC)-conjugated anti-human secondary antibody (Sigma-Aldrich, St. Louis, MO, USA) for 20 min at 4 °C. After being washed three times, the pellets containing the bound cot-HER2apt-MMAEs were resuspended in binding buffer. Fluorescence was examined using a BD FACSCalliburTM and FACSVerseTM system (BD Biosciences, San Jose, CA, USA) with 10,000 events being counted. The data were analyzed using FlowJo software v10.0.7.

### 4.4. Confocal Imaging

To evaluate the internalization of aptamers, NCI-N87 cells (3 × 10^4^) were plated onto Nunc Lab-Tek II Chambered Coverglass and incubated at 37 °C for 48 h. After washing twice with pre-warmed Dulbecco’s PBS, the cells were incubated in binding buffer at 37 °C for 2 h, and simultaneously treated with 100 pmol of 5′- cyanine 5 (Cy5)-conjugated HER2 aptamers (cy5-HER2apts) and 5 μM DAPI. DAPI is a fluorescent dye used to stain nuclear DNA of the living cells. Images of live cells on the CO_2_ chamber were collected using an LSM 780 confocal microscope (Carl Zeiss, Oberkochen, Germany).

### 4.5. Cell Proliferation Assay

Cell proliferation was measured using a WST-1 Cell Proliferation Assay Kit (Takara, Kyoto, Japan) according to the manufacturer’s instructions. In brief, NCI-N87 cells (1 × 10^4^) were seeded into 35 mm plates and cultured 24 h in RPMI containing 1% FBS. The cells were treated with serial dilutions of MMAE, VC-MMAE, or cot-HER2apt-MMAEs. After incubation for 6 d, 10 μL of WST-1 reagent was added to each well and the cells were incubated for an additional 2 h at 37 °C. The proportion of surviving cells was determined by measuring the absorbance of the formazan product at 450 nm and reference absorbance at 650 nm using a SpectraMax Plus plate reader (Molecular Devices, Sunnyvale, CA, USA).

### 4.6. Nude Mouse Xenograft Model of Gastric Cancer

Seven-week-old female BALB/c-nude mice were purchased from Orient Bio Inc. (Seongnam, Korea). The animals were housed under specific pathogen-free conditions and acclimated to the laboratory conditions for at least 1 week before use. The mice were maintained in the animal facility of the National Cancer Center, which is an accredited unit of the National Cancer Center Research Institute (unit number NCC-16-342), in accordance with the AAALAC International Animal Care Policy.

The xenograft model of gastric cancer was established by subcutaneous injection of NCI-N87 cells (1 × 10^7^) into the flanks of the nude mice. When the tumors reached approximately 200 mm^3^ in volume, the animals were intravenously injected with PBS, control HER2-DOligobody (HER2aptctl; reverse sequence of HER2apt14), HER2-DOligobody (14 mer), or HER2-DOligobody (28 mer) twice a week for 2 weeks. The HER2-DOligobodies injections contained 1.27 mg/kg cot-HER2apt-MMAEs pre-incubated in 10 mg/kg cot-body. Tumor volumes were measured every 3 d or 4 d using digital calipers (Mitutoyo, Utsunomiya, Japan) and total volumes calculated as cubic millimeters using the formula (length × width^2^)/2, where length was the longest axis and width was the distance perpendicular to the length.

To assess systemic toxicity, the body weight (BW) of the mice was monitored weekly. After 36 d, animals were euthanized, and blood samples collected. The blood samples were centrifuged at 7000 rpm for 20 min at 4 °C, and the sera were stored at −80 °C for subsequent evaluation of biochemical parameters. Serum levels of glutamic oxaloacetic transaminase (GOT), glutamic pyruvic transaminase (GPT), blood urea nitrogen (BUN), creatinine (CRE) and total bilirubin (TBIL) were measured using a Fuji Dri-Chem 3500 biochemistry analyzer (Fujifilm, Tokyo, Japan).

### 4.7. Statistical Analyses

All statistical analyses were performed using GraphPad Prism software (GraphPad Software Inc., San Diego, CA, USA). Differences between two groups were assessed using the Student’s *t*-test, and a *p*-value < 0.05 was considered statistically significant.

## 5. Conclusions

In the current study, we developed DOligobody, a drug-conjugated oligobody composed of an aptamer—drug conjugate and antibody. We demonstrated that the conjugate takes advantage of beneficial aspects of both the aptamers and ADCs. Our DOligobody strategy may overcome the current concerns of ADCs, regarding the difficulty of modulating site-specific conjugation, their low stability, and limited tissue penetration. Furthermore, systemic administration of aptamers is not effective due to rapid renal clearance, but the DOligobody has the potential to be effective because of its high stability in vivo. The DOligobody platform also has the advantage that it can be easily reassembled with a desired aptamer, so it can block multiple target molecules/pathways in cancer cells. Overall, the results of this study suggest that DOligobodies demonstrate strong advantages for their use in anti-cancer therapeutics.
